# Spatio-Temporal Proximity Characteristics in 3D μ-Printing via Multi-Photon Absorption

**DOI:** 10.3390/polym8080297

**Published:** 2016-08-10

**Authors:** Erik Hagen Waller, Georg von Freymann

**Affiliations:** 1 Physics Department and Research Center OPTIMAS, University of Kaiserslautern, 67663 Kaiserslautern, Germany; georg.freymann@physik.uni-kl.de; 2 Fraunhofer-Institute for Physical Measurement Techniques (IPM), 67663 Kaiserslautern, Germany

**Keywords:** 3D μ-printing, direct-laser-writing, photo resist, polymerization

## Abstract

One of the major challenges in high-resolution μ-printing is the cross-talk between features written in close proximity—the proximity effect. This effect prevents, e.g., gratings with periods below a few hundred nanometers. Surprisingly, the dependence of this effect on space and time has not thoroughly been investigated. Here, we present a spatial-light-modulator based method to dynamically measure the strength of the proximity effect on length and timescales typical to μ-printing. The proximity strength is compared in various photo resists. The results indicate that molecular diffusion strongly contributes to the proximity effect.

## 1. Introduction

Direct laser writing (DLW) lithography—or 3D μ-printing via multi-photon absorption—is the most versatile technology for rapid prototyping of three-dimensional (3D) micro structures exploiting two-photon absorption (TPA) [1]. Having seen a huge push of its benchmarks in recent years, DLW has become the enabling technology for a broad range of structures including the fields of photonics, micro optics, micro biology, and micro mechanics [2]. However, while feature sizes of direct laser written structures may almost arbitrarily be reduced, reducing feature separations still remains challenging [3]. Features written in close proximity influence each other, causing undesired effects such as linewidth broadening, tiny sporadic connections and bending [4,5]. In their entity, these observations are termed ‘proximity effect’. A number of mechanisms could potentially contribute to the proximity effect including polymer chain growth, scattering of light, thermal or molecular diffusion, and dose addition during subsequent illumination steps. The mechanisms underlying the proximity effect are, however, neither precisely known nor is the effect’s strength and temporal evolution well characterized. Thus, we introduce here a new method to quantitatively characterize the proximity effect’s strength on a spatial- and temporal scale typical to DLW. To this end, we make use of the flexibility of a spatial-light-modulator (SLM) based DLW system. Including the SLM into the DLW setup allows for accurate spatial beam shaping and as a consequence for point-spread-function engineering, permitting, e.g., arbitrarily placed multi foci (MF) [6,7]. The suggested method exploits such specifically positioned MF to study the mutual influence of features in close proximity, as described in the experiments and methods section. The results not only give new insight into the spatial and temporal extent of the proximity effect in DLW but also indicate that molecular diffusion strongly influences feature sizes on comparably large length and time scales.

## 2. Materials and Methods

**Setup:** The SLM-based DLW setup differs from the conventional DLW setup by the introduction of an SLM, which is imaged by a 4-*f* setup onto the entrance pupil of the objective. Details on the SLM-based DLW setup may be found in [6].

**MF pattern:** The algorithm computing the phase pattern, which, in turn, leads to the desired MF is based on a weighted Gerchberg–Saxton algorithm that takes into account high numerical aperture effects. Details on this algorithm may be found in [7].

**Substrate preparation:** 170 μm thick round glass substrates with a diameter of 30mm are cleaned in two subsequent ultra-sonic bath steps (each 45kHz, 10min), first in aceton, second in isopropanol. Afterwards, substrates are rinsed with water and blow-dried.

**Resist application:** IP-L is drop-cast onto the substrate. IP-G is drop-cast onto the substrate and pre-baked for 1 h at $100{\;}^{{}^{\circ}}C$. SU-8 50 is spin-coated at 3000rpm for 30s to yield a 40μm thick film. It is then soft-baked for 6min at $65{\;}^{{}^{\circ}}C$ followed by 15min at $95{\;}^{{}^{\circ}}C$. For SU-8, a post-exposure bake is necessary: 1min at $65{\;}^{{}^{\circ}}C$ followed by 4min at $95{\;}^{{}^{\circ}}C$. All bakes are done on a contact hot-plate.

**Development:** IP-L is developed by immersion of the sample in isopropanol for 20min and subsequent rinsing with water. IP-G is developed by immersion in AZ–EBR (AZ Electronic Materials GmbH, Wiesbaden, Germany) for 20min. SU-8 is developed by immersion in AZ–EBR for 6min. All samples are gently blow-dried.

**Scanning electron micrographs:** Before taking the micrographs, samples are sputtered with 3nm Iridium. A SEM (SU8000, Hitachi High-Technologies Corporation, Tokyo, Japan) is used to take the micrographs.

**Figure 1:**
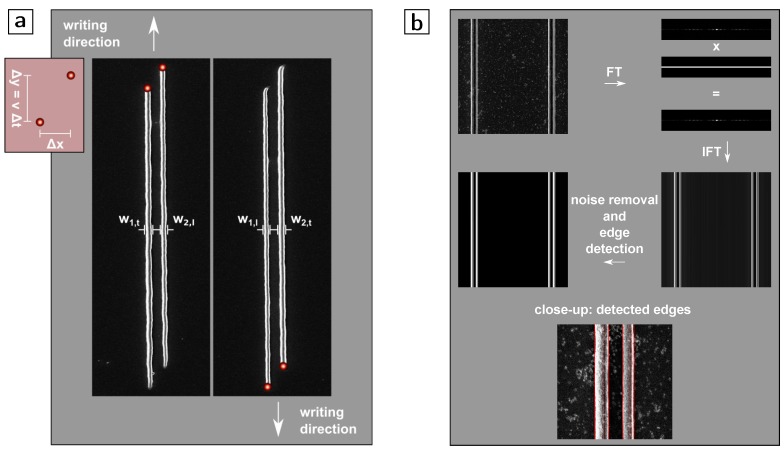
(**a**) Inset: scheme of the arrangement of the two foci. Displacement of the foci orthogonal to the writing direction by Δx and along the writing direction by Δy. One focus trails the other in time by Δt=Δy/v with *v* being the writing speed. Main figure: Exemplary scanning electron micrographs of lines written in alternating writing directions with such a multi foci arrangement (indicated schematically). The linewidths *w* are defined and labelled according to their relative position (left focus →1, right focus →2, trailing along the writing direction →t and leading along the writing direction →l). All linewidths are found by automatic edge detection from micrographs and are individually averaged over a length of several micrometers along the middle of each line (the latter avoids non-steady-state conditions); and (**b**) edge detection method: scanning electron micrographs (pixel size 7nm) are Fourier transformed, filtered in the Fourier domain and back transformed. Subsequently, remaining noise is removed and edges are detected by a steepest-slope approach.

**Edge detection and fitting:** From the micrographs, the edges of the lines are automatically determined by Fourier transformation, filtering for edge enhancement and back transformation (also see Figure 1b). The fitting is computed by minimization of the mean-squared error.

## 3. Experimental Results

Details on the SLM-based DLW setup may be found elsewhere [6]. In short, the output of a Ti:Sa laser (Chameleon Ultra 2, Coherent Inc., Santa Clara, CA, USA) is guided to overfill the aperture of a SLM (X10468-02, Hamamatsu Photonics, Hamamatsu, Japan). The SLM modulates the wavefront and—by exploiting changes in diffraction efficiency—the intensity of the laser beam. The thus obtained pattern is circularly polarized and imaged onto the aperture of a high numerical aperture objective (numerical aperture NA=1.4, Plan-ApoChromat DIC, Carl Zeiss Microscopy GmbH, Jena, Germany) by a 4-f-setup. The required phase and intensity patterns that, as a consequence, lead to the desired MF, are calculated by a method published in [7]. Alternatively, a blazed grating may be displayed on the SLM to mimic conventional DLW.

To ease the interpretation of the results, we restrict ourselves to patterns that produce two foci with various relative positions. The two foci are positioned in a way where one focus trails the other along the writing direction by Δy and where the foci are separated by Δx orthogonal to the writing direction (see the inset of Figure 1 for a sketch).

Two lines are simultaneously written by each foci pair (Figure 1a). For most experiments, we keep the writing speed constant at v=100 μm/s and may therefore calculate the delay between the foci by Δt=Δy/v. For each foci pattern, the incident laser power is adjusted such that the line written by the leading focus measures approximately 250nm in width. It is thus kept far below the damage threshold but likewise well enough above the polymerization threshold to enable stable lines. Note that both the damage threshold as well as the polymerization threshold depend on the dose per area and thus on the foci arrangement. Average powers of the light incident on the entrance pupil of the objective are in the range of a few milliwatts. The negative-tone photo resists IP-L (radical-initiated, liquid, Nanoscribe GmbH, Eggenstein-Leopoldshafen, Germany) , IP-G (radical-initiated, gel-like, Nanoscribe GmbH, Eggenstein-Leopoldshafen, Germany) and SU-8 50 (cation-initiated, solid, micro resist technology GmbH, Berlin, Germany) are processed as usual (see Materials and Methods section for details) and used in this comparative study. As in conventional DLW, structures are revealed by a development step.

After the development step, for each foci pair, the linewidths of the corresponding two lines are measured by automatic edge detection using scanning electron micrographs (Figure 1b). Since absolute linewidths alone are not necessarily an indicator for the proximity effect, we use the relative broadening of the delayed line (which experiences an already affected photo resist) compared to leading line (which experiences an unaffected photo resist). To this end, the widths are related by dividing the width of the line written by the trailing focus by the width of the line written by the leading focus: $b=w_{t}/w_{l}$. Since the peak intensity of the two foci is not exactly identical (see reference [7]), the writing direction is reversed and *b* is averaged over the two directions ($b=1/2\left(w_{1,t}/w_{1,l}+w_{2,t}/w_{2,l}\right)$, where 1,2 accounts for the first (left in the figure) and second line (right in the figure), respectively, and l,t for the leading and trailing focus, see Figure 1). This procedure cancels out effects that stem from deviations in the foci’s intensity uniformity and leaves us with only the relative effect one line has on the other. In addition, compared to using absolute linewidths, this procedure has the advantage of cancelling one fitting constant in the diffusion model discussed below.

Note that our method requires a minimum separation Δx of approximately 400nm for separated lines. Therefore, we cannot exclude that further mechanisms contribute to the proximity effect at even smaller separations as obtainable, e.g., in stimulated emission depletion lithography. Furthermore, some potential uncertainties due to shrinking, bending and tilting of the lines as well as slightly different focus positions with respect to substrate surface lead to some experimental noise (values of *b* differ ±0.025 across different but identically parallelly written structures).

### 3.1. Polymerization Reaction

In this section, we present the experimental results on the spatial and temporal dependencies of the proximity effect for various photo resists. We also include the proximity effect’s dependence on writing speed and laser power, and compare the proximity effect in parallel DLW using MF with serial DLW using a single focus. The photo resists are chosen to represent members that undergo radical chain polymerization as well as cationic ring-opening polymerization. The qualitative interpretation of the observations requires some knowledge of the two polymerization reactions, which we shortly review here.

For type I radical chain polymerization, it is generally accepted that structure formation proceeds in the following simplified steps [8]:

1. Photo initiator molecules two-photon absorb incident radiation and subsequently cleave into radicals.
2. The generated radicals bind to monomer molecules, thus forming a propagation radical.
3. The propagation radical continues to bind to monomer molecules until the chain reaction is terminated by inhibitor molecules (mostly oxygen) or a second radical.

In total, a well-crosslinked polymer network evolves anywhere where a sufficient large number of radicals is created. Oxygen plays an important role in radical chain polymerization, as it acts as a quencher for the excited photo initiator molecules as well as an inhibitor for the chain reaction [9]. This ‘oxygen barrier’, as well as a minimum crosslinking density to withstand the development process, lead to a threshold behaviour of the photo resist where only above a certain threshold radical generation rate a solid structure evolves.

In contrast, the cation ring-opening polymerization proceeds as follows [10]:

1. Photo initiator molecules two-photon absorb incident radiation and subsequently cleave into ions. In case of SU-8, H^+^ cations are the initiating species.
2. The generated ions open ring-molecules ionizing the latter.
3. The ionized molecule continues to open rings until the reaction is terminated by transfer reactions or bimolecular interaction with other species, e.g., water.

For a better understanding of the experimental results, two things are worth mentioning regarding the ring-opening polymerization: first, the H^+^ ion is much smaller than any of the rather complex radicals in radical-based polymerization, and thus more mobile, and second, oxygen does not influence the reaction. Therefore, we expect large differences in the spatio-temporal characteristics of the proximity effect in the two polymerization reactions.

### 3.2. Proximity in IP-G (Gel-Like, Radical-Initiated)

**Figure 2:**
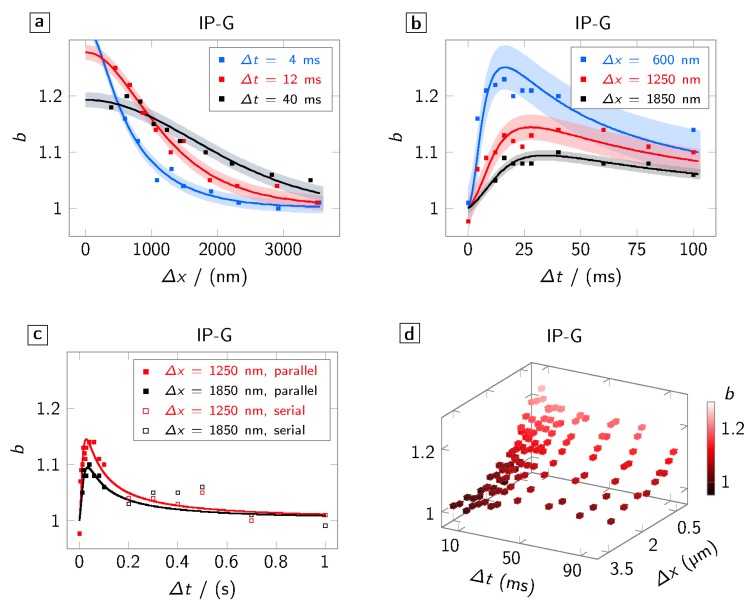
Spatio-temporal characteristics of the broadening $b=w_{t}/w_{l}$ in the gel-like, radical based photo resist IP-G. Squares indicate the measured data, the solid line a model fit to the experimental values, and the underlay the corresponding mean-squared error. (**a**) Spatial characteristics of *b* for exemplary delays Δt; (**b**) temporal characteristics of *b* for exemplary separations Δx; (**c**) comparison of temporal characteristics of *b* in serial (open squares) and parallel (filled squares) writing mode; and (**d**) spatio-temporal characteristics of *b*.

We begin by discussing the spatial scale of the proximity effect in the gel-like, radical-initiated IP-G photo resist. For these experiments, the separation Δx of the two foci is varied while keeping the delay Δt constant. Figure 2a shows the broadening $b=w_{t}/w_{l}$ over Δx for various time delays Δt. Hereby, squares indicate measured values, the solid line a radical diffusion model discussed in the next section fitted to the measured data, and the correspondingly coloured underlay the mean-squared error. For all Δt shown in the graph, a non-negligible broadening is observed on spatial scales that extend well beyond what is expected by the focal intensity distribution (FWHM: ≈400nm). This already indicates that the linewidth is not solely defined by the exciting squared intensity distribution.

Looking at the dependence of the broadening on the delay Δt, we notice a quick rise to a maximum value within a few milliseconds (Figure 2b) and a slow decay within several hundreds of milliseconds (Figure 2c). Hereby, the maximum depends on the separation and is shifted towards longer delays for larger separations. Since clipping in the optical path does not allow for the separation between the two foci to be larger than 10 μm (corresponding to 100ms delay), for longer delays, we use a single focus with a well-defined waiting time approach. For the serial writing scheme, one has to take into account that, due to the longer time span, substrate drifts and thus measurement errors are more likely to occur. This leads to a lager error of *b* than ±0.025 (see, e.g., the kink at 0.5s). Taking this consideration into account, the values obtained in this scheme follow the natural extrapolation of the parallel writing scheme. This hints that the effect neither depends on the writing mode (serial or parallel) nor—since, in serial mode, no change in phase pattern is necessary—on the phase pattern displayed on the SLM. In fact, the overall spatio-temporal trend (Figure 2d) of the broadening effect indicates that a diffusive process is the underlying mechanism.

**Figure 3:**
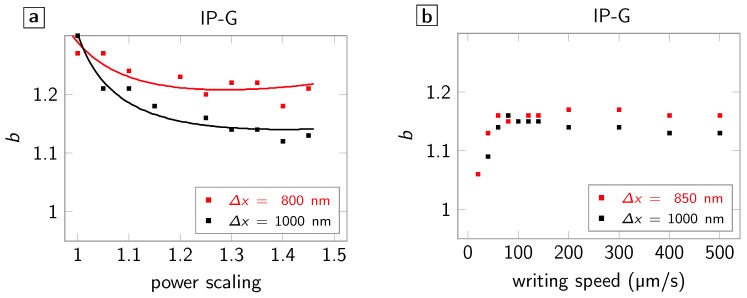
Writing power and speed dependence of the broadening $b=w_{t}/w_{l}$ in IP-G. Squares indicate the measured data. The solid lines are trend lines given as a guide to the eye. (**a**) Dependence of *b* on incident laser power; and (**b**) dependence of *b* on writing speed.

To determine further characteristics of the broadening effect, we investigate its dependencies on incident laser power and on writing speed (Figure 3). Figure 3a shows the dependence of the broadening on the incident laser power. We hereby chose a reference laser power that guarantees stable lines (approximately 20% above the threshold power for simultaneous polymerization by two foci separated by 600nm) but is still well below the damage threshold, which is approximately 100% above the reference power. We define power scaling as incident power over reference power. A slight reduction of *b* over this large (1.5 times the reference power) power increase is observed—in line with the expectation that any contribution by diffusion to the linewidth becomes negligible when large laser powers are used. We may therefore conclude that small power fluctuations typically observed in DLW only affect *b* to a negligible extent.

Regarding the dependence on writing speed (Figure 3b), we see a drop of *b* towards small writing speeds and a rather constant behaviour above ≈60 μm/s all the way up to 500 μm/s, which is the maximum writing speed that we can test in this setup while keeping Δt constant at 20 ms. This observation, too, may be explained by a diffusion process: When the writing speed becomes slower than the diffusion of, e.g., molecules, the linewidth is more and more influenced by molecules diffusing along the writing direction and less so by molecules diffusing from the position of the other focus. Furthermore, when approaching a writing speed of zero, there is no priority of any of the two foci and a broadening of one is expected. Contrary, when the writing speed is larger than the diffusion of the molecules, the opposite is the case and a constant value for *b* is expected for a given Δt. Note that this finding is especially relevant for galvanometer-based DLW systems that typically reach writing speeds of several tens of millimetres per second. While, potentially, several lines may be written with these systems before this effect becomes relevant, the effect does not vanish, leading to broader features written later in time. Thus, structures written at these writing speeds are affected in a more complex manner than nearest neighbour interaction. In total, a final structure will be slightly asymmetric (especially when shrinkage during development is taken into account) with respect to the starting point of the writing process, unless sophisticated writing strategies are taken into account that consider the length and timescales found in this study.

### 3.3. Comparison with IP-L (Liquid, Radical-Initiated)

**Figure 4:**
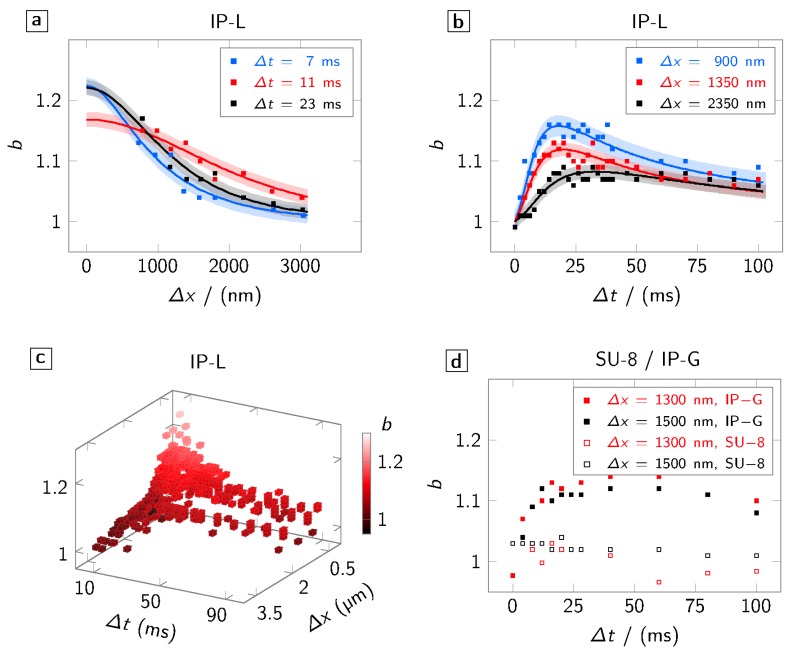
Spatio-temporal characteristics of the broadening $b=w_{t}/w_{l}$ in the liquid, radical based photo resist IP-L. Squares indicate the measured data, the solid line a model fit to the experimental values, and the underlay the corresponding mean-squared error. (**a**) Spatial characteristics of *b* for exemplary delays Δt; (**b**) temporal characteristics of *b* for exemplary separations Δx; (**c**) spatio-temporal characteristics of *b*; and (**d**) comparison of the temporal characteristics of *b* in SU-8 (open squares) and IP-G (filled squares) for exemplary values of Δx.

IP-L is used as a comparable photo resist that undergoes a similar polymerization reaction upon illumination as IP-G. It differs, however, in its viscosity since it is a liquid photo resist. Again, we evaluate the spatial (Figure 4a), the temporal (Figure 4b) and the spatio-temporal (Figure 4c) characteristics of the proximity effect. Fundamentally similar diffusive spatio-temporal characteristics of *b* are observed in this liquid photo resist as in IP-G. The decay over time after the maximum of *b* is reached, however, seems to be somewhat more pronounced. This could be an indication for a larger diffusion constant in the liquid resist compared to the more solid version.

### 3.4. Comparison with SU-8 (Solid, Cation-Initiated)

Finally, we investigate the broadening effect in the cationic-initiated photo resist SU-8 50 and compare it to values obtained in the radical-based photo resists. The temporal characteristic in Figure 4d clearly shows that hardly any—if any—broadening is observed for the former on the time scales under study. The absence of the effect in SU-8 may be explained either by the insensitivity of the cationic-initiated polymerization to the presence (and diffusion) of oxygen or by different time scales on which the small (compared to the initiators in radical-initiated polymerization) photo acids diffuse.

### 3.5. Interpretation

The experimental data gives evidence that a diffusive process is the underlying cause of the broadening *b*. By ruling out other possibilities, we here deduce that, in fact, molecular diffusion is responsible for the spatio-temporal characteristics of *b*.

In the introduction, we already mentioned potential mechanisms that could contribute to the proximity effect. These include polymer chain growth outside the exposed area as well as absorption, scattering and diffraction of light by intermediate reaction products or already polymerized structural details. Furthermore, fluorescence, non-radiative energy transfer mechanisms, dose addition via crosslinking density increase during subsequent illumination steps, and, finally, diffusion of heat or molecules with the latter potentially combined with oxygen depletion. The vast majority of these contributions are, however, negligible on the spatial and temporal scales studied in the present work.

Fluorescence occurs much faster in the two radical based photo resists than it could explain the temporal characteristic of the broadening. Furthermore, supported by reference [11] and Monte-Carlo simulations conducted in our group (not shown), we may safely assume the polymerization reaction to have mostly terminated before about 2ms (if we assume an exposure time of less than 4ms due to the writing speed being 100μm/s and the full-width-half maximum of the excitation focus being 380nm). Therefore, any structure–light interaction should lead to a non-vanishing temporal characteristic of *b* after a few milliseconds. Since this is not the case—the broadening effect completely vanishes over time—these interactions may be ruled out to be the cause. The same argument holds true for dose addition and polymer growth. Non-radiative energy transfer mechanisms are mostly due to heat and could potentially contribute to the broadening effect. However, in reference [12], the local temperature increase in ultra-short pulse DLW is measured and found to be only relevant when using very high illumination intensities. Since here we use low to moderate intensities (slightly above the threshold), heat transfer and heat diffusion may also be dismissed.

In conclusion, this leaves us with molecular diffusion of either initiation radicals, propagation radicals and/or inhibitors (oxygen), which is studied in depth in the following section.

## 4. Numerical Results

To quantitatively interpret the experimental results, we need to find a model that predicts the linewidth of a direct laser written feature.

In a first simplification, we may assume that a well crosslinked structure forms anywhere where a certain radical generation rate threshold $R_{\mathrm{th}}$ is exceeded. The threshold rate may be explained by the minimum number of radicals that need to be created to either form a well crosslinked polymer network or to counteract inhibition by, e.g., oxygen. In TPA, the radical generation rate is proportional to the squared intensity distribution, which we may assume to follow a Gaussian distribution (we restrict the discussion to one dimension):

(1) $$R\left(x\right)=R_{0}\exp\left[-4\frac{x^{2}}{\sigma^{2}}\right]\;,$$

where $R_{0}$ corresponds to the maximum rate and *σ* determines the width of the distribution.

The effective radical generation rate may, however, also be increased or decreased respectively by radicals or inhibitors (mainly oxygen) diffusing into the excited volume (diffusion rate $R_{\mathrm{diff}}$):

(2) $$R\left(x\right)=R_{0}\exp\left[-4\frac{x^{2}}{\sigma^{2}}\right]+R_{\mathrm{diff}}\;.$$

wherever $R\left(x\right)\geq R_{\mathrm{th}}$, a solid polymer network forms. Therefore, at R(w/2), the equality holds (with *w* being the linewidth). Thus, we may predict the linewidth by:

(3) $$\begin{array}{ccc} w & = & \sqrt{-\sigma^{2}\ln\left[\frac{R_{\mathrm{th}}-R_{\mathrm{diff}}}{R_{0}}\right]}, \end{array}$$

(4) $$\begin{array}{ccc} & = & \sqrt{-\sigma^{2}\left(\ln\left[\frac{R_{\mathrm{th}}}{R_{0}}\right]+\ln\left[1-\frac{R_{\mathrm{diff}}}{R_{\mathrm{th}}}\right]\right)}\;. \end{array}$$

These equations apply to either diffusing radicals or diffusing inhibitors. For further interpretation of the results, however, we deduce distinct diffusion models for the two potentially diffusing substances. We concentrate here on the diffusion model for radical diffusion and leave the model for inhibitor diffusion to Appendix A.

### Diffusion of Radicals

To further simplify the above expression, we now make some assumptions on the values of $R_{\mathrm{th}}$ and $R_{\mathrm{diff}}$. The contribution by diffusion $R_{\mathrm{diff}}$ is small compared to $R_{\mathrm{th}}$ making the term $R_{\mathrm{diff}}/R_{\mathrm{th}}$ very small. In total, the argument of the second logarithm under the square root is close to one. We may, therefore, approximate: $\ln\left[1-R_{\mathrm{diff}}/R_{\mathrm{th}}\right]\approx -R_{\mathrm{diff}}/R_{\mathrm{th}}$. Factoring out the first logarithm and merging all constant terms into two different constants: α≥0 and β≥0, in total, the linewidth may be calculated by:

(5) $$w\approx \sqrt{\alpha \left(1+\beta R_{\mathrm{diff}}\right)}\;.$$

**Figure 5:**
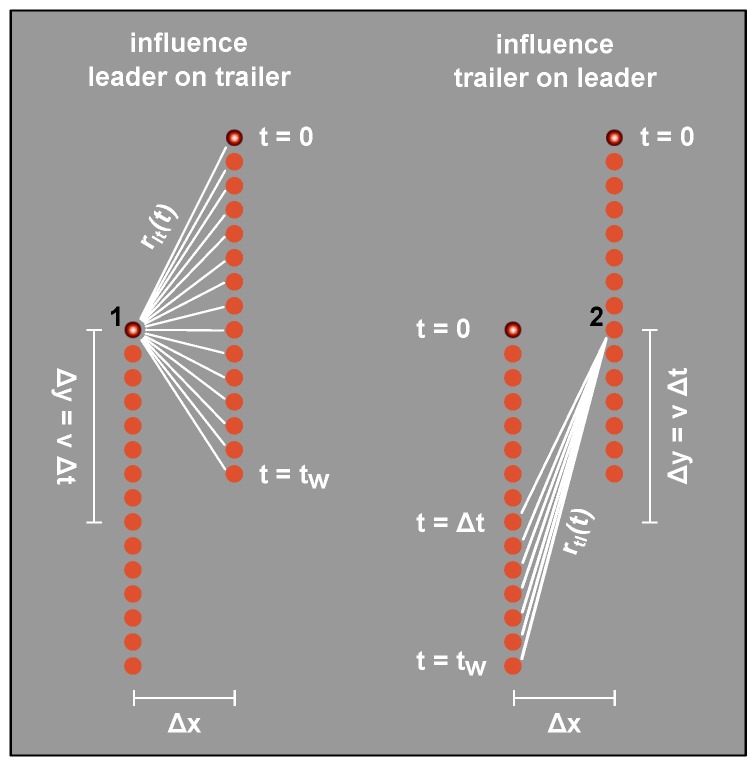
Radical diffusion scheme. **Left**: the leading line affects the trailing line. Radicals diffuse from various positions on the leading line (**red** circles) to position 1 (marked by the **black** 1). The **white** lines denote the diffusion distance which changes over time. The lines indicate diffusion prior to the trailing spot reaching position 1. It follows from the figure: $r_{\mathrm{lt}}^{2}\left(t\right)=\Delta x^{2}+{\left(\Delta y-vt\right)}^{2}$ (*v* is the writing speed). $t_{w}$ is the total writing time. **Right**: the same as the left but indicating how the trailing line affects the leading line. Here, we find: $r_{\mathrm{tl}}^{2}\left(t\right)=\Delta x^{2}+{\left(vt\right)}^{2}$.

Following the above considerations, if we neglect inhibitor diffusion, in its most simplified form, the broadening $b=w_{t}/w_{l}$ is given by:

(6) $$b=\frac{w_{t}}{w_{l}}=\sqrt{\frac{1+\beta R_{\mathrm{diff},\;l\to t}^{\left(\mathrm{rad}\right)}}{1+\beta R_{\mathrm{diff},\;t\to l}^{\left(\mathrm{rad}\right)}}}\;,$$

where l→t denotes the total contribution by radicals created by the leading focus to the radical generation rate and vice versa (see Figure 5).

These contributions may be calculated: from Figure 5, we see that the momentary total amount of radicals present at position 1 when the trailing focus passes may be described as the sum over contributions from point sources diffusing into half-space (and vice versa) [13]. Therefore:

(7) $$\begin{array}{ccc} R_{\mathrm{diff},\;l\to t}^{\left(\mathrm{rad}\right)} & \propto & \int_{0}^{t_{w}}\frac{1}{4{\sqrt{\pi Dt^{\prime }}}^{3}}\exp\left[-\frac{\Delta x^{2}+{\left(\Delta y-vt^{\prime }\right)}^{2}}{4Dt^{\prime }}\right]dt^{\prime }\;, \end{array}$$

(8) $$\begin{array}{ccc} R_{\mathrm{diff},\;t\to l}^{\left(\mathrm{rad}\right)} & \propto & \int_{\Delta t}^{t_{w}}\frac{1}{4{\sqrt{\pi Dt^{\prime }}}^{3}}\exp\left[-\frac{\Delta x^{2}+{\left(vt^{\prime }\right)}^{2}}{4Dt^{\prime }}\right]dt^{\prime }\;, \end{array}$$

with *D* being the effective diffusion constant and with the other parameters being defined in Figure 5.

We emphasize that, in total, this model only contains two fitting constants: *β* and *D*. Also note that, in this derivation, we assume only a dependence on the momentary amount of radicals since—due to the smaller molecule size—we may expect inhibitor diffusion to occur tremendously faster than radical diffusion. Thus, we may consider inhibitor numbers to be constant, and no contribution of radicals that have passed through over time is expected.

## 5. Discussion

**Figure 6:**
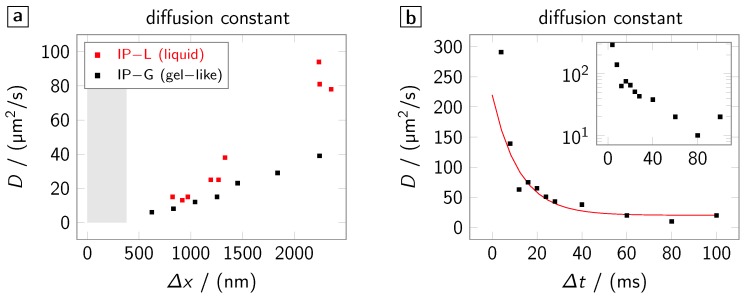
Effective diffusion constant determined from the fits. (**a**) Dependence of the constant on the line separation in IP-L and IP-G. The **grey** area indicates the spatial extent (full-width-half-maximum) of the excitation focus; and (**b**) dependence of the constant on the time delay in IP-G. The **red** line is an exponential fit to the data excluding the values at 4ms and 100ms. The inset shows a log-plot of the data.

The radical diffusion models described in the previous section are fitted to the experimental data using mean-squared error minimization (the fits are presented in Figure 2 and Figure 4). This allows for extracting the effective diffusion constant *D* in dependence on either Δx or Δt. This information is provided in Figure 6. Hereby, Figure 6a shows the dependence of *D* on Δx for the photo resists IP-G and IP-L. In both resists, the effective diffusion constant reduces with smaller line separations. This is intuitively clear since, at small separations, the gap between the lines ‘sees’ a comparatively high intensity and, therefore, partly polymerizes. Thus, with reduced line separation, the effective diffusion constant approaches the diffusion constant in already polymerized material while, at large separations, it approaches the one in a non-polymerized resist. This interpretation is backed by the fact, that for both the gel-like (IP-G) and the liquid (IP-L) resist the diffusion constant approaches approximately the same value for small line separations but diverges at large separations with the diffusion constant being larger in the liquid resist. Furthermore, the order of magnitude of the diffusion constant found by our method is in agreement to estimations of the diffusion constants of propagation radicals given in literature [14].

Figure 6b shows the temporal dependence of the effective diffusion constant in IP-G. The temporal evolution of D mostly follows an exponential trend. This is in accordance with free volume theory where the diffusion constant of growing polymer chains (propagation radicals) is found to decrease exponentially with increasing molecular weight of the chains [15]. Since Monte-Carlo simulations (not shown) reveal that, at the above millisecond time scale considered here, monomer addition and thus the molecular weight increase is almost linear over time, this exponential behaviour is expected. The liquid character of IP-L leads to stronger line distortions as mentioned above causing more experimental noise and linewidth errors. Therefore, for IP-L, the exponential trend is not as clear and is thus excluded from the figure.

The excellent agreement of the model developed here with the experimental observations plus the close correspondence of the diffusion constants obtained here with values found in literature is a strong indication that diffusion of radicals is indeed the cause of the broadening observed in DLW. However, we may not completely dismiss oxygen diffusion to be the underlying mechanism (refer to the supporting material). Thus, further experiments need to be conducted to pinpoint the major contributor, radicals and / or oxygen, to the proximity effect.

## 6. Conclusions

In conclusion, we exploit parallelized DLW to study the mutual influence of structural features in DLW on a millisecond time- and micrometer spatial scale. The measurements give indication that molecular diffusion either by radicals or oxygen strongly affects the material response. Hereby, the response extends well beyond the reaction volume and increases the linewidth on a spatial scale on the order of several micrometers and on a temporal scale of several hundreds of milliseconds.

Two models, one describing the influence of diffusion of propagation radicals and one describing the influence of diffusion of oxygen on structural features, are introduced. Both models individually may explain the spatio-temporal characteristics of the linewidth broadening and lead to diffusion constants on the order of several tens of μm^2^/s. Further experiments are necessary to distinguish between radical and oxygen diffusion.

## Figures and Tables

**Figure A1:**
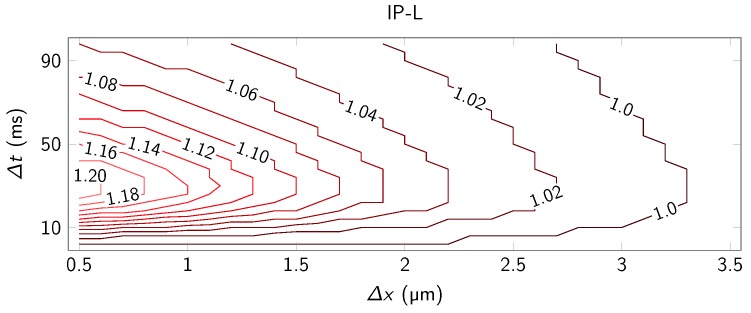
Oxygen diffusion model fitted to the experimental data. The calculation is done on a discretized grid and no smoothing is applied.

## Appendix A

The data presented in the main body of this paper does not allow for dismissing inhibitor diffusion as the underlying cause for the proximity effect. To describe the effect which inhibitor (e.g., oxygen) diffusion might have on the radical chain reaction, we follow a slightly different reasoning compared to the radical diffusion model. However, we again restrict our discussion to only one diffusing substance and one dimension.

The inhibition rate is proportional to the inhibitor concentration and increases the effective threshold rate. Thus, similar to Equation (3) in the main paper, we obtain:

(A1) $$w=\sqrt{-\sigma^{2}\ln\left[\frac{R_{\mathrm{th}}+R_{O_{2}}}{R_{0}}\right]}\;,$$

with $R_{O_{2}}$ being the threshold increase by oxygen that is proportional to the oxygen concentration: $R_{O_{2}}=\gamma_{1}C_{O_{2}}$. The broadening *b* then becomes:

(A2) $$b=\sqrt{\frac{\ln\left[\frac{R_{\mathrm{th}}+\gamma_{1}C_{O_{2},1}}{R_{0}}\right]}{\ln\left[\frac{R_{\mathrm{th}}+\gamma_{1}C_{O_{2},2}}{R_{0}}\right]}}\;.$$

It is now left to determine the inhibitor concentration, which depends on the time delay Δt between, and the spatial separation Δr of, the two foci: during the polymerization reaction—which terminates within a few milliseconds [11]—inhibitor molecules are consumed. Thus, the inhibitor concentration $C^{\left(i\right)}$ is quickly reduced in a large volume surrounding the excitation volume. This volume is an interaction volume that affects all post illumination steps.

The closest the leading focus gets to a position exposed by the trailing focus is Δr. When assuming a Gaussian dependence on Δr, we may thus approximate the inhibitor concentration at this position by ($\gamma_{2}$ being a constant):

(A3) $$C_{2}^{\left(i\right)}=C_{0}^{\left(i\right)}\left(1-\gamma_{2}\cdot \exp\left[-\frac{\Delta x^{2}+\Delta y^{2}}{\sigma^{\prime 2}}\right]\right)\;.$$

On the contrary, the closest separation between the trailing focus and the line exposed by the leading focus is Δx, and, therefore, the trailing focus “sees” a much lower inhibitor concentration. However, during the time Δt, inhibitor molecules diffuse back into the interaction volume. The trailing focus thus experiences the following inhibitor concentration:

(A4) $$C_{1}^{\left(i\right)}\left(\Delta t\right)=C_{0}^{\left(i\right)}\left(1-\gamma_{2}\cdot \exp\left[-\frac{\Delta x^{2}}{\sigma^{\prime 2}}\right]\right)+C_{\mathrm{diff}}^{\left(i\right)}\left(\Delta t\right)\;,$$

where $C_{\mathrm{diff}}^{\left(i\right)}$ accounts for the concentration change due to diffusing inhibitor molecules.

The concentration $C_{1}^{\left(i\right)}$ may be approximated by diffusion from a large volume with constant concentration $C_{0}^{\left(i\right)}$ into a sphere with concentration $C_{1}^{\left(i\right)}\left(0\right)$. At the center of the sphere, $C_{1}^{\left(i\right)}$ is then calculated by [13]:

(A5) $$C_{1}^{\left(i\right)}\left(\Delta t\right)=C_{0}^{\left(i\right)}+2C_{\beta }\exp\left[-\frac{\Delta x^{2}}{\sigma^{\prime 2}}\right]\sum_{n=1}^{\infty }{\left(-1\right)}^{n}\exp\left[-Dn^{2}\pi^{2}\Delta t/a^{2}\right]\;,$$

where *a* is the radius of the sphere. Inserting $C_{1}^{\left(i\right)}$ and $C_{2}^{\left(i\right)}$ into Equation (A2), we obtain:

(A6) $$b=\sqrt{\frac{\ln\left[\beta_{1}+2\beta_{2}\exp\left[-\frac{\Delta x^{2}}{\sigma^{\prime 2}}\right]\sum_{n=1}^{\infty }{\left(-1\right)}^{n}\exp\left[-Dn^{2}\pi^{2}\Delta t/a^{2}\right]\right]}{\ln\left[\beta_{1}-\beta_{2}\exp\left[-\frac{\Delta x^{2}+\Delta y^{2}}{\sigma^{\prime 2}}\right]\right]}}\;,$$

where we incorporated all terms that do not depend on Δx and Δy in the constants $\beta_{1,2}$. Note that this model leaves us with five fitting constants, $0<\beta_{1}=\left(R_{\mathrm{th}}+\gamma_{1}C_{0}\right)/R_{0}<1$, $0<\beta_{2}=\gamma_{1}C_{\beta }/R_{0}\leq \beta_{1}$, $\sigma^{\prime }$, *D*, and *a*.

Figure A1 shows a fit of the oxygen diffusion model to the experimental values of *b* in IP-L. Hereby, the sum was taken over 10,000 elements and different initial guesses for the parameters were used for the error minimization algorithm. The obtained fit constants ($\beta_{1}=0.5$, $\beta_{2}=0.175$, a=5.05μm, $\sigma^{\prime }=1.67\;\mu m$, $D=55.6\;\mu m^{2}/s$) are within their limits and physically reasonable. While the overall agreement of this model with the measured data depends very much on the initial guesses for the parameters and is less accurate than the radical diffusion model, it points in the right direction and should therefore be kept in mind for future experiments.
